# Of the Extermination of the Small-Pox

**Published:** 1803-04-01

**Authors:** 


### *Of the* Extermination *of the* Small-Pox.

THE loathsome appearance of the natural Small-pox, as well as the really dangerous nature of the disease, must always have rendered an attack of that malady an object of terror to mankind; and if we refer to the days of Sydenham and of Morton, we shall find that a large share of the emoluments of the medical profession was derived from the treatment of this disease. The introduction of inoculation tended considerably to diminish those emoluments. But although the Faculty cannot boast of the honour of introducing that important Discovery, they warmly interested themselves in promulgating it, and cheerfully sacrificed more selfish and interested considerations, in order to promote the general welfare of man kind.

But the liberal behaviour of the Faculty on that occasion, bears no comparison with their present disinterested conduct. Inoculation did not wholly deprive the Small-pox of its terrific features, or of its danger. The practice of inoculation still produced a handsome income to the practitioners of physic, though it might be divided among a larger number of individuals, or diffused through an inferior sphere of the profession. But to attempt the utter annihilation of the Small-pox, breathes a higher tone of disinterested benevolence; it exhibits, indeed, a singular and surprising example of a sacrifice of self interest, at the shrine of public virtue. Not less astonishing, than if we were to behold the whole respectable corps of counsellors, attornies, and bailiffs, laying their wigs, their heads I mean, together, to form such a code of pure, equitable, and intelligible laws, as would in future enable mankind to live in concord and amity, and supersede the necessity of all legal proceedings in the common courts of justice.

The eagerness and enthusiasm, nearly equal to that of the antient crusaders, with which medical men have propagated this discovery, are not easily to be accounted for. In its day, the discovery of inoculation was, perhaps, equal in importance to that of the Vaccinia; but there was no such fury in forcing it down the throats of mankind. People were left to the freedom of their own choice, and inoculation was diffused in proportion to the good sense and discernment of mankind. Being somewhat inclined to suspect the disinterestedness of all men, when it is opposed by their interest, I am apt to think that medical men were afraid lest the practice of Vaccination should slip through their fingers, and were therefore in haste to reap all the profitable fruits of this discovery, ere mankind were aware that their interference was unnecessary. It is evident indeed, that the communication of this happy discovery must depend on its being taken out of the hands of the Faculty. The Small-pox will never be extirpated till every parent is in the habit of inserting the vaccinia in the arm of his child, as regularly as they carry it to church to be christened. *Apropos!* How would it answer to employ the clergy in this benevolent purpose, and have both the operations performed at the same time? It would have given a stronger proof of professional disinterestedness to have seen plans circulated containing plain directions for parents to inoculate their own children, than to behold not only individual *persons*, but even *circles* of medical men, puffing off the safety of the Cow-pock in the public prints, and themselves obliquely as the only beings who knew how to manage it.

Having thus given the Faculty due credit for the purity and disinterestedness of their conduct on the present occasion, let us shortly enquire what beneficial effects their new practice is likely to have in a political point of view. That as great a number of mankind exist at all times, and in all places, as can procure the food they wish, is a point I believe generally agreed upon by every enlightened writer on political economy; or, if any doubts on this subject remained, they have been compleatly removed by Mr. Malthas in his late truly Philosophical Essay on the Principle of Population. That gentleman has decidedly proved, that while population proceeds in a geometrical ratio, or by multiplication, the utmost efforts of the industry of mankind cannot augment the means of their subsistance faster than in an arithmetical ratio, or by addition, and that of course there is, at all times, a superfluity of population compared with the means of existence. To effect a due equilibrium between these two opposing forces, appears to be one purpose of the introduction of vice and misery into the system of the world; the former tending to prevent, and the latter to diminish the too rapid increase of mankind. Disease is a species of misery; and as nations grow old population increases, while the natural fertility of the soil diminishes in a more rapid ratio than the labours of men can augment its productive powers; new diseases spring up to thin, from time to time, the ranks of mankind, and equalize the principles of generation and production. Among this class of the instruments of Providence, must the Small-pox be reckoned; while syphilis must be classed among the list of preventives. Neither of these diseases were known to the antients. How well the Small-pox has performed its part, is proved by the fact, that the numbers who die of that disease, are more now than before the introduction of inoculation. Among that class of society, indeed, who could afford to be at the expence and trouble of having their children inoculated, the hideous scars of the disease are less frequently met with, and the appearance of the human face divine is improved, but the average number of persons who perish by the disease has not been diminished among the lower, the more numerous class of society; among that class of whom society may be said, in fact, to consist, its ravages have been undiminished.

If the necessity of these equalizing forces be allowed, and no person who has perused the above Treatise can doubt it, is there not reason to apprehend some mischief from attempting to throw any one of them out of action? When the gradual and unheeded action of disease, and of infant mortality, become inadequate to check the progress of the increasement of mankind, have we not reason to dread that war, famine, or pestilence, those awful ministers of heaven's wrath, may become necessary to rectify the idle interference of medling man.

Examples of such mistaken policy are not wanting. In a district of America, the inhabitants took it into their heads that a peculiar species of pigeon destroyed their crops. The thunder of the gun never ceased till they were wholly exterminated. But in proportion as their numbers were diminished, so was the produce of the soil. When too late, it was discovered that these useful animals preyed upon the insects which, in fact, destroyed the grain; and in order to enable them to reap their usual harvests, they were obliged, with considerable trouble, to renew the breed of their feathered friends. In this country, till very lately, a similar mistake prevailed respecting moles. They were supposed to injure vegetation, and therefore universally persecuted; whereas, in fact, they are carnivorous animals, preying on the slug, the great enemy of horticulture.

I might farther illustrate this point, by shewing that disease, and particularly epidemics, are rare in colonies and new settled countries, but augment with the age of society; for nations have their diseases as well as individuals, and at length length destroy peculiar aggregations of mankind; as is the case in Egypt and Africa, once the most flourishing parts of the world, now almost depopulated, and in great measure by the effects of small-pox, measles, and pestilence, which from thence have extended their ravages to other parts of Europe; but I have already trespassed too long upon your time.

I humbly presume to think, that medical men would do as well to confine themselves to their own sphere, and endeavour to benefit those individuals who applied to them for assistance, letting the welfare of mankind find its own level. The art of legislation is out of their line. It has been well defined by a poet, who himself honoured the fraternity as a member. Virgil describes his physician as one who

> Scire potestates herbarum, usumque medendi
> Maluit; et *mutas* agitare *inglorius* artes.

Benevolence, or public good, too often serves men, and sometimes I believe without their being conscious of it, as a stalking-horse, to hide their own interested motives.

These considerations, in the mind of the writer, militate strongly against the sanguine hopes of the amelioration of the condition of man, expected by some from the practice of the Cow-pock; and they apply with tenfold force against the establishment of it by law.* He may, however, be wrong, and is open to conviction; if any of your Correspondents will deign to enlighten his mind, it will confer a favour on, Yours,

SCEPTICUS.

---

### *A* CASE *of* THORACIC ADHESIONS; *communicated by* Mr. M. F. WAGSTAFFE, *of Southwark.*

ON the 16th of January, 1802, I was requested to attend the daughter of Mr. Morris, of the Borough, about fourteen years of age, thin spare habit, and sallow complexion, but of an active disposition, labouring under a severe attack of rheumatismus acutus, evidently characterised by

---

* We do not believe that any person ever thought of establishing Vaccination by law. We are no more friendly to *such* legislative interference than S. himself; but we observe that all governments occasionally do, and we think they ought, to attend still more to the *health* of the people. ED.

